# Loss, Gain and Altered Function of GlyR α2 Subunit Mutations in Neurodevelopmental Disorders

**DOI:** 10.3389/fnmol.2022.886729

**Published:** 2022-04-29

**Authors:** Xiumin Chen, Katie A. Wilson, Natascha Schaefer, Lachlan De Hayr, Mark Windsor, Emmanuel Scalais, Germaine van Rijckevorsel, Katrien Stouffs, Carmen Villmann, Megan L. O’Mara, Joseph W. Lynch, Robert J. Harvey

**Affiliations:** ^1^Queensland Brain Institute, The University of Queensland, Brisbane, QLD, Australia; ^2^Research School of Chemistry, The Australian National University, Canberra, ACT, Australia; ^3^Institute of Clinical Neurobiology, University Hospital, Julius-Maximilians-University Würzburg, Würzburg, Germany; ^4^School of Health and Behavioural Sciences, University of the Sunshine Coast, Maroochydore, QLD, Australia; ^5^Sunshine Coast Health Institute, Birtinya, QLD, Australia; ^6^Neurologie Pédiatrique, Centre Hospitalier de Luxembourg, Luxembourg, Luxembourg; ^7^Center for Medical Genetics, Universitair Ziekenhuis Brussel, Vrije Universiteit Brussel, Brussels, Belgium; ^8^Australian Institute for Bioengineering and Nanotechnology (AIBN), The University of Queensland, Brisbane, QLD, Australia

**Keywords:** autism spectrum disorder, developmental disorders, epilepsy, glycine receptor (GlyR), *GLRA2*, GlyR α2 subunit

## Abstract

Glycine receptors (GlyRs) containing the α2 subunit govern cell fate, neuronal migration and synaptogenesis in the developing cortex and spinal cord. Rare missense variants and microdeletions in the X-linked GlyR α2 subunit gene (*GLRA2*) have been associated with human autism spectrum disorder (ASD), where they typically cause a *loss-of-function* via protein truncation, reduced cell-surface trafficking and/or reduced glycine sensitivity (e.g., *GLRA2*Δex8-9 and extracellular domain variants p.N109S and p.R126Q). However, the GlyR α2 missense variant p.R323L in the intracellular M3-M4 domain results in a *gain-of-function* characterized by slower synaptic decay times, longer duration active periods and increases in channel conductance. This study reports the functional characterization of four missense variants in *GLRA2* associated with ASD or developmental disorders (p.V-22L, p.N38K, p.K213E, p.T269M) using a combination of bioinformatics, molecular dynamics simulations, cellular models of GlyR trafficking and electrophysiology in artificial synapses. The GlyR α2^V–22L^ variant resulted in altered predicted signal peptide cleavage and a reduction in cell-surface expression, suggestive of a *partial loss-of-function*. Similarly, GlyR α2^N38K^ homomers showed reduced cell-surface expression, a reduced affinity for glycine and a reduced magnitude of IPSCs in artificial synapses. By contrast, GlyR α2^K213E^ homomers showed a slight reduction in cell-surface expression, but IPSCs were larger, with faster rise/decay times, suggesting a *gain-of-function*. Lastly, GlyR α2^T269M^ homomers exhibited a high glycine sensitivity accompanied by a substantial leak current, suggestive of an *altered function* that could dramatically enhance glycinergic signaling. These results may explain the heterogeneity of clinical phenotypes associated with *GLRA2* mutations and reveal that missense variants can result in a loss, gain or alteration of GlyR α2 function. In turn, these GlyR α2 missense variants are likely to either negatively or positively deregulate cortical progenitor homeostasis and neuronal migration in the developing brain, leading to changes in cognition, learning, and memory.

## Introduction

Glycine receptors (GlyRs) are key mediators of synaptic inhibition in the retina, inner ear, and throughout the developing brain, brainstem, and spinal cord (Malosio et al., 1991; Wässle et al., 2009; Buerbank et al., 2011). GlyRs form part of a superfamily of ligand-gated ion channels that includes inhibitory GABA_A_, GABA_C_ and excitatory nAChR and 5HT_3_ receptors. These ion channels have a common pentameric receptor configuration and for GlyRs can be formed from either homomeric α or heteromeric αβ subunit conformations (Lynch, 2004). Although the exact subunit stoichiometry of heteromeric GlyRs has been a matter of extensive debate, recent cryo-electron microscopy studies of native GlyRs have strongly suggested a 4α:1β arrangement, with inclusion of multiple β subunits rendering the receptor non-conductive (Yu et al., 2021; Zhu and Gouaux, 2021). Each GlyR subunit has an N-terminal signal peptide (SP), a large N-terminal domain (NTD) that mediates subunit assembly and ligand-binding (Du et al., 2015; Huang et al., 2015; Yu et al., 2021; Zhu and Gouaux, 2021) and four membrane-spanning domains (M1–M4), followed by a short extracellular C-terminus. The M3–M4 intracellular loop differs among GlyR subunits and provides sites for interactions with accessory proteins such as collybistin (Breitinger et al., 2020), gephyrin (Sola et al., 2004; Kim et al., 2006), and syndapin-1 (Langlhofer et al., 2020), as well as opportunities for subunit-specific modulation by G-protein-coupled receptor-mediated signaling pathways linked to GlyR phosphorylation (Harvey et al., 2004; Manzke et al., 2010).

Five genes encoding distinct GlyR subunits have been characterized in rodents and humans: *GLRA1*-*GLRA4* and *GLRB*, encoding the GlyR α1-4 and β subunits, respectively. GlyRs containing the α1 subunit are pivotal in spinal motoneuron inhibition, and consistent with this role, dominant and recessive mutations in the GlyR α1 and β subunit genes are associated with a rare neurological disorder known as startle disease/hyperekplexia (OMIM 149400; 614618; 614619; Shiang et al., 1993; Rees et al., 2002; Chung et al., 2010, 2013; Bode et al., 2013; James et al., 2013; Piro et al., 2021). This disorder affects newborn humans, dogs, horses, and cattle (Harvey et al., 2008) and is characterized by an exaggerated startle response and muscle hypertonia in response to unexpected acoustic, tactile or visual stimuli. In humans, dominant missense mutations in the GlyR α1 subunit typically disrupt the transduction pathway linking ligand-binding to ion-channel gating, while recessive mutations in the GlyR α1 and β subunits result in ligand-binding or protein trafficking deficits (Villmann et al., 2009; Chung et al., 2010; Bode et al., 2013; James et al., 2013; Schaefer et al., 2015; Piro et al., 2021). Although these dominant and recessive mutations result in an overall loss of GlyR function, other more complex disease pathomechanisms also exist. For example, the GlyR α1^P366L^ mutant disrupts interactions with syndapin 1, an F-BAR domain protein involved in membrane remodeling (Langlhofer et al., 2020). Moreover, a subset of GlyR α1 and β subunit mutations result in a gain or alteration of function (Chung et al., 2010, 2013; Bode et al., 2013; James et al., 2013; Zhang et al., 2016; Piro et al., 2021). For example, some missense mutations increase the sensitivity to glycine (α1^I43F^), prolong the decay rate of inhibitory postsynaptic currents (IPSCs; α1^I43F^, α1^W170S^, α1^Q266E^, α1^V280M^, α1^R414H^), or induce spontaneous GlyR channel opening (α1^I43F^, α1^Y128C^, α1^W170S^, α1^Q226E^, α1^V280M^, α1^R414H^, β^L285R^). GlyRs containing the α3 and α4 subunits have not yet been linked to human disease, but studies using knockout and knockin mice have implicated GlyR α3 in central pain sensitization (Harvey et al., 2004; Werynska et al., 2021), rhythmic breathing (Manzke et al., 2010), and ethanol-mediated behaviors (Blednov et al., 2015; San Martin et al., 2021). GlyR α4 is a pseudogene in humans but contributes to touch-evoked escape behaviors in zebrafish (Leacock et al., 2018) and impacts embryonic development and litter sizes in rodents (Nishizono et al., 2020).

Glycine receptors containing the α2 subtype were initially assigned key roles in synaptogenesis, with GlyR activation resulting in membrane depolarization, triggering local opening of L-type Ca^2+^ channels that resulted in clustering of gephyrin and GlyR at developing postsynaptic sites (Kirsch and Betz, 1998; Lévi et al., 1998). However, it rapidly became apparent that the kinetic properties of GlyR α2 are inconsistent with a synaptic function, since GlyR α2 exhibits slow activation kinetics (Mangin et al., 2003) and activates for longer durations than other GlyR subtypes (Krashia et al., 2011). Consistent with this, non-synaptic taurine or glycine release onto GlyRs was found to be vital for neocortical and spinal cord development (Flint et al., 1998; Scain et al., 2010). Studies using *Glra2* knockout mice have since revealed pivotal roles for GlyR α2 in retinal photoreceptor development (Young and Cepko, 2004) and the control of receptive field surrounds in retinal ganglion cells (Nobles et al., 2012; Zhang et al., 2015a), as well as modulation of ethanol intake, aversion and preference (Blednov et al., 2015; San Martin et al., 2020; Araya et al., 2021). However, a major role for GlyR α2 has been identified in dorsal cortical progenitor homeostasis and cortical interneuron migration (Avila et al., 2013, 2014). Extrasynaptic activation of GlyR α2 in cortical interneurons by endogenous glycine activates voltage-gated Ca^2+^ channels, which modulates actomyosin contractions to fine-tune nuclear translocation during interneuron migration (Avila et al., 2013). Knockout of GlyR α2 disrupts cortical progenitor homeostasis, impairing the capacity of apical progenitors to generate basal progenitors resulting in an overall reduction of projection neurons in upper or deep layers of the cerebral cortex (Avila et al., 2014). As a result, moderate microcephaly was observed in newborn mice (Avila et al., 2014). Further studies of *Glra2* knockout mice revealed permanent effects on the mature cortical networks: somatosensory cortical neurons had more dendritic branches with an overall increase in total spine number, resulting in an overall increase network excitability and enhanced susceptibility to epileptic seizures after pentylenetetrazol (PTZ) injections (Morelli et al., 2017). *Glra2* knockout mice also exhibited defects in long-term potentiation in the prefrontal cortex and object recognition memory (Pilorge et al., 2016) and impaired motor memory consolidation (Molchanova et al., 2018).

These findings led to the exploration of the X-linked human GlyR α2 subunit gene (*GLRA2*) as a candidate gene for childhood neurological disorders associated with cortical or cognitive defects. *GLRA2* defects were indeed reported in individuals with autism spectrum disorder (ASD; Pinto et al., 2010; Piton et al., 2011; Iossifov et al., 2014; Pilorge et al., 2016), although additional clinical symptoms were reported in some cases, including delay/loss of acquired language and seizures (Piton et al., 2011; Pilorge et al., 2016). For example, a microdeletion (*GLRA2*Δex8-9) and two *de novo* missense mutations p.N109S and p.R126Q (p.N136S and p.R153Q in the GlyR α2 subunit with signal peptide) were found in the hemizygous (XY) state in males (Pilorge et al., 2016). The microdeletion *GLRA2*Δex8-9 produced a truncated GlyR α2 subunit protein lacking M3, the cytoplasmic M3–M4 intracellular loop and M4 that was not expressed at the cell surface. By contrast, two missense mutations, GlyR α2^N109S^ and α2^R126Q^, caused reduced cell-surface expression and loss of glycine sensitivity (Pilorge et al., 2016). A third missense variant in *GLRA2* (p.R323L), associated with autism, macrocephaly, seizures and hypothyroidism in a female proband (Piton et al., 2011), was found to result in a gain of function (Zhang et al., 2017). Electrophysiological analysis of GlyR α2^R323L^ revealed slower synaptic decay times, longer duration of active periods and an increase in conductance of α2^R323L^ and α2^R323L^β channels (Zhang et al., 2017). In this study, we provide insights into the functional properties of four novel missense variants in *GLRA2* associated with ASD and developmental disorders (p.V-22L, p.N38K, p.K213E, p.T269M) using a multidisciplinary approach, encompassing cellular models of GlyR trafficking, molecular modeling, and electrophysiology using artificial glycinergic synapses. These variants cause either loss, gain or altered function of GlyR α2, explaining the range of clinical presentations associated with *GLRA2* variants.

## Materials and Methods

### Molecular Biology, Bioinformatics and Molecular Modeling

The majority of the GlyR α2 variants studied were sourced from published sources (Iossifov et al., 2014; Krumm et al., 2015; Deciphering Developmental Disorders Study, 2017. GlyR α2 p.K213E was identified by ES, GvR, and KS in diagnostic exome sequencing. Site-directed mutagenesis of the human GlyR α2 subunit cDNA in the expression vector pRK5 (Zhang et al., 2017) was performed using the QuikChange kit (Agilent, Santa Clara, CA, United States). The successful incorporation of mutations was confirmed by Sanger DNA sequencing, performed by DNA Sequencing and Services (MRC PPU, School of Life Sciences, University of Dundee, United Kingdom) and analysis using Sequencher software (Gene Codes, Ann Arbor, MI, United States). Plasmid DNAs were prepared using a HiSpeed Plasmid Maxi Kit (QIAGEN, Hilden, Germany). The damaging effects of human GlyR α2 subunit variants were assessed using Sorting Intolerant From Tolerant (SIFT; Sim et al., 2012), PolyPhen-2 (Adzhubei et al., 2013), and CADD scores (Rentzsch et al., 2019). Mutation position in the GlyR α2 subunit is indicated using mature subunit numbering (i.e., after signal peptide cleavage). The effects of the V-22L variant on signal peptide cleavage were assessed using SignalP 4.0 (Petersen et al., 2011). Molecular modeling of the p.N38K and p.K213E variants was accomplished using the recently resolved structures of the GlyR α2β pentamer in the closed (RCSB: 7L31) and glycine-bound open state (RCSB: 5BKF) (Yu et al., 2021). GlyR structures were visualized using the UCSF ChimeraX molecular visualization program (Pettersen et al., 2021). Amino acid substitutions were modeled using the swapaa command, taking into account the highest rotamer prevalence (Dunbrack backbone-dependent rotamer library, Shapovalov and Dunbrack, 2011), the highest number of H-bonds and the lowest clash score.

### Molecular Dynamics Simulations

A homology model of the GlyR α2 homopentamer was also constructed using the Phyre2 web server (Kelley et al., 2015). GlyR α2 monomers were overlaid onto the GlyR α1 homopentamer (PDB ID: 3JAE; Du et al., 2015) to create the GlyR α2 homopentamer. The GlyR α2^T269M^ variant was created by introducing M269 into the GlyR α2 homopentamer using PyMOL (DeLano, 2002). Wild-type and GlyR α2^M269^ homopentamer systems were embedded into a model membrane composed of 80% POPC and 20% CHOL. Each system was solvated with SPC water, neutralized with Na^+^ and NaCl was added to a concentration of 0.15 M. This led to an overall system consisting of the GlyR α2 homopentamer, in a membrane containing 80 mol% POPC and 20 mol% CHOL, surrounded by ∼155,000 water molecules and 0.15 M NaCl. Simulations were conducted in the apo state, in the absence of the ligand glycine. All systems were simulated using GROMACS 2019.4 molecular dynamics engine (Abraham et al., 2015) in conjunction with the GROMOS 54a7 force field (Schmid et al., 2011). The system was energy minimized using the steepest descent algorithm and equilibrated in five sequential 1 ns simulations with decreasing restraints on the protein (1000 kJ mol^–1^ nm^–1^, 500 kJ mol^–1^ nm^–1^, 100 kJ mol^–1^ nm^–1^, 50 kJ mol^–1^ nm^–1^, and 10 kJ mol^–1^ nm^–1^). Each unrestrained system was then simulated in triplicate for 500 ns. In all simulations, a 2 fs timestep was used. The pressure was maintained at 1 bar using semi-isotropic pressure coupling using the Berendsen barostat (τ_P_ = 0.5 ps and isothermal compressibility = 4.5 × 10^–5^ bar), and the temperature was maintained at 300 K using the Bussi-Donadio-Parrinello velocity rescale thermostat (τ_T_ = 0.1 ps). Periodic boundary conditions were implemented. SETTLE was used to constrain the geometry of water molecules and LINCS was used to constrain the covalent bond lengths of the solute. Analysis was performed using the GROMACS tools and the trj_cavity package on the entire 1.5 μs of combined production simulation for each system. The Visual Molecular Dynamics (VMD) program was used for visualization of the simulations (Humphrey et al., 1996; Paramo et al., 2014).

### Cell-Surface Trafficking Assays

HEK293 cells (CRL-1573; ATCC – Global Biosource Center, Manassas, VA, United States) were transfected with GlyR constructs using the Ca^2+^ phosphate-DNA co-precipitation method as previously described (Sontheimer et al., 1989). Cells were washed 6 h post- transfection and used for biotinylation assays (New England Biolabs, Ipswich, MA, United States) 48 h after transfection. Biotin labeling and subsequent binding to streptavidin was used to discriminate between whole-cell and cell-surface protein. Surface proteins were labeled by incubating the cells for 30 min with 1 mg/ml EZ-Link Sulfo-NHS-LC-Biotin [sulfosuccinimidyl-6-(biotinamido)hexanoate (Pierce Biotechnologies, Rockford, IL, United States)]. Following a quenching step (192 mM glycine, 25 mM Tris in PBS, pH 8.0 for 10 min), cells were detached by using ice-cold PBS buffer. After centrifugation for 10 min at 1,000 × *g*, cells were lysed with TBS (Tris-buffer saline with 1% Triton-X100 and protease inhibitor mixture tablet, Roche Diagnostics, Mannheim, Germany). After centrifugation for 1 min at 13,000 × *g*, the supernatant (whole protein fraction) was incubated with 50 μl of streptavidin-agarose beads (Pierce Biotechnologies, Rockford, IL, United States) for 2 h at 4°C. Beads were washed three times in TBS buffer. Biotinylated proteins (surface fraction) were eluted by boiling with 50 μl of 2 × SDS buffer for 5 min at 95°C. Whole cell (WC) and cell surface (SF) fractions were separated by SDS-PAGE and Western blotting on nitrocellulose membranes (GE Healthcare, Little Chalfont, United Kingdom). Membranes were blocked for 1 h with 5% BSA in TBS-T (TBS with 1% Tween 20). GlyR α2 subunits were detected with the antibody mAb4A (cat. no. 146011, 1:1,000, Synaptic Systems, Göttingen, Germany). Pan-cadherin (Cell Signaling Technology, Danvers, MA, United States, 4068, 1:1000) served as a loading control for the whole-cell fraction and cell-membrane protein fractions. Signals were detected using the ECL plus system (GE Healthcare, Little Chalfont, United Kingdom). Image quantification was performed using ImageJ (1.51)/Fiji2 (Schindelin et al., 2012, 2015; Schneider et al., 2012). Data were analyzed using Student’s *t*-test. For all tests, the number of asterisks corresponds to the level of statistical significance: *p < 0.05; ^**^p < 0.01; ^***^p < 0.001. Values are displayed as means ± standard error of the mean (±SEM) unless otherwise noted.

### Primary Culture of Spinal Neurons

Spinal neurons were prepared using methods as previously described (Dixon et al., 2015; Zhang et al., 2015b,^2017^; Leacock et al., 2018). Briefly, E15 timed-pregnant rats were euthanized via CO_2_ inhalation in accordance with procedures approved by The University of Queensland Animal Ethics Committee (Approval number: QBI/142/16/NHMRC/ARC). The spinal cords were rapidly removed, triturated and plated onto poly-D-lysine-coated coverslips in a 4-well plate at a density of 8–10 × 10^4^ cells/well, and cultured for 3–4 weeks until spontaneous inhibitory postsynaptic currents (IPSCs) could be detected. The cells were initially cultured in Dulbecco’s Modified Eagle’s Medium (DMEM) supplemented with 10% fetal bovine serum (DMEM-FBS). After 24 h, the entire DMEM-FBS medium was replaced with Neurobasal medium including 2% B27 and 1% GlutaMAX supplements. A second and final feed 1 week later replaced half of this medium with fresh Neurobasal medium. Neurons were used in co-culture experiments between 1 and 4 weeks later.

### HEK293 Cell and Artificial Synapse Preparations

Artificial synapses were generated as previously described (Dixon et al., 2015; Zhang et al., 2015b,^2017^; Leacock et al., 2018). Briefly, HEK293 cells were cultured in DMEM-FBS until ∼90% confluent. One day prior to transfection, cells were trypsinized and plated onto glass coverslips in 35 mm culture dishes at a density of 5 × 10^3^ cells/dish. Each dish was transfected with 0.3 μg of GlyR α2 subunit DNA, plus 0.1 μg EGFP (pEGFP) was used as a transfection marker. For artificial synapses, 0.3 μg of mouse neuroligin 2A (pNice) and 0.3 μg of rat gephyrin (pCIS) were also added. Transfection was performed via a Ca^2+^ phosphate-DNA co-precipitation method for 15–20 h in a 3% CO_2_ incubator and terminated by washing cells twice with divalent cation-free phosphate buffered saline. Cells were trypsinized the next day, centrifuged and re-suspended in Neurobasal medium (including 2% B27 and 1% GlutaMAX supplements) then seeded onto neurons. One 35 mm dish of HEK293 cells was typically sufficient to seed four coverslips of neurons. Once seeded with HEK293 cells, the co-cultures were returned to the incubator overnight to allow artificial synapses to form between neurons and transfected HEK293 cells. Cells were used for patch-clamp recording over the following 2–3 days.

### Electrophysiology

Whole-cell patch clamp recordings were performed at room temperature (22 ± 1°C). Glycine concentration-response relationships were performed at −40 mV, whereas artificial synapse recordings were performed at −70 mV, both using a MultiClamp 700B amplifier and pCLAMP 10 software (Molecular Devices). Signals were filtered at 4 kHz and sampled at 10 kHz. Patch pipettes (4–8 MΩ resistance) were fabricated from borosilicate glass (GC150F-7.5, Harvard Apparatus) and filled with an internal solution comprising (in mM): 145 CsCl, 2 CaCl_2_, 2 MgCl_2_, 10 HEPES, and 10 EGTA, adjusted to pH 7.4 with CsOH. The extracellular solution comprised (in mM) 140 NaCl, 5 KCl, 2 CaCl_2_, 1 MgCl_2_, 10 HEPES, and 10 D-glucose, adjusted to pH 7.4 with NaOH.

### Electrophysiology Data Analysis

Analyses of IPSC amplitudes, 10–90% rise times, and decay time constants were performed using AxoGraph X (AxoGraph Scientific). Only cells with a stable series resistance of <25 MΩ throughout the recording period were selected for analysis. IPSCs were detected using a semi-automated sliding template. Each detected event was visually inspected and only those with no inflections in the rising or decay phases were included. All selected events from a single cell were digitally averaged. Parameters derived from these digitally averaged waveforms were then pooled with those from other cells to obtain group data. To calculate macroscopic current decay time constants, digitally averaged macroscopic recordings were fitted with double-exponential functions in AxoGraph X, and a weighted time constant was calculated from individual time constants (τ1, τ2) and their relative amplitude (A1, A2) as follows: τ_weighted_ = (τ1 × A1 + τ2 × A2)/(A1 + A2). Displayed averaged data represent group means ± SEMs. The Hill equation was used to calculate the saturating current magnitude (I_max_), half-maximal concentration (EC_50_), and Hill coefficient (n_H_) values for glycine activation. Individual concentration-response relationships were fitted using a non-linear least squares algorithm (SigmaPlot 11.0; Jandel Scientific, San Rafael, CA, United States). Statistical analysis and graphing were performed with SigmaPlot 11.0. Data were first tested for normality using both the Shapiro–Wilk and Kolmogorov–Smirnov tests. Via either test, all data proved normally distributed using an alpha value of 0.05. Statistical analysis was then performed using a one-way ANOVA for multiple comparisons followed by Tukey’s *post hoc* test. *P* values of <0.05 were taken to be statistically significant.

## Results

### Identification of Candidate *GLRA2* Variants and Bioinformatic Analysis

**FIGURE 1:**
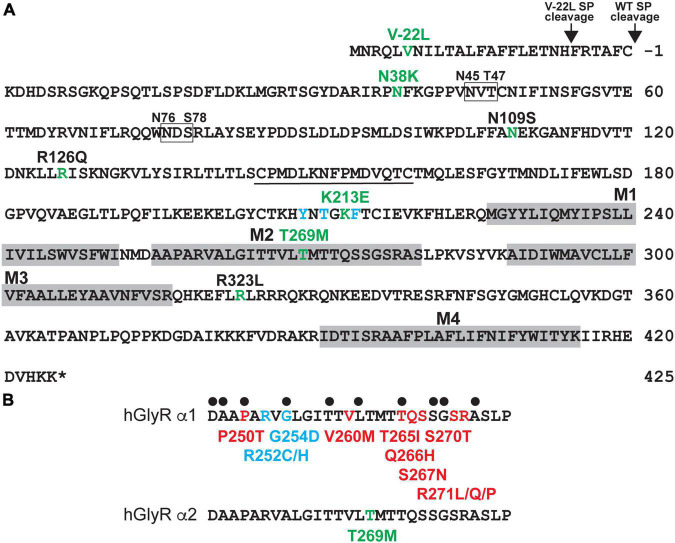
Human GlyR α2 subunit variants associated with ASD and neurodevelopmental disorders. **(A)** Human GlyR α2 subunit missense variants p.V-22L (signal peptide), p.N38K (ECD), p.K213E (ECD), and p.T269M (M2) were identified in individuals with autism spectrum disorder (ASD) or developmental disorders (DD). Amino acid sequence of the human GlyR α2 subunit indicating the positions of putative membrane-spanning domains (gray shaded boxes), amino-acid residues affected by missense changes associated with ASD or DD (green), key glycine-binding residues (blue), and glycosylation sites (boxes). **(B)** Alignment of the human GlyR α1 and α2 subunits showing pore-lining residues (black dots) and different types of pathogenic mutations found in the GlyR α1 subunit in human startle disease. Red, dominant; blue, recessive; green, spontaneously opening channels (leakage current). Note that GlyR α2^T269M^ does not affect predicted pore-lining residue, nor does it correspond to a position of a known GlyR α1 subunit startle disease mutation.

Candidate GlyR α2 subunit mutations were identified from exome sequencing studies in ASD and/or developmental disorders (Iossifov et al., 2014; Krumm et al., 2015; Deciphering Developmental Disorders Study, 2017; Figure 1A and Supplementary Table 1) and include p.V-22L (signal peptide), p.N38K (extracellular domain, ECD), p.K213E (ECD), and p.T269M (M2). These correspond to substitutions p.V6L, p.N65K, p.K240E, and p.T296M in the human GlyR α2 subunit precursor prior to signal peptide cleavage. The damaging effects of the human GlyR α2 subunit variants were assessed using SIFT (Sim et al., 2012), PolyPhen-2 (Adzhubei et al., 2013), and CADD scores (Rentzsch et al., 2019; Supplementary Table 1). All variants were found to be possibly/probably damaging with PolyPhen-2 and all had high CADD scores: p.V-22L, 15.78; p.N38K, 20.4, p.K213E, 24.3 and p.T269M 25.4, consistent with previously reported GlyR α2 subunit variants associated with ASD (p.N109S, p.R126Q, p.R323L; Supplementary Table 1). All variants were absent from gnomAD database (Karczewski et al., 2020) with the exception of p.K213E, which occurs with a low frequency of 4/177,746 alleles. However, given the high CADD score for p.K213E (24.3), we proceeded with structure/function analysis. The variant p.V-22L would not normally be expected to affect GlyR α2 subunit function, since it is located in the cleavable signal peptide found at the N-terminus of the protein. However, on analysis with SignalP 4.0 (Petersen et al., 2011) we noted that p.V-22L subtly alters the predicted signal peptide cleavage site for GlyR α2. While the wild-type protein was predicted to be cleaved between amino acids 27 and 28 AFC-KD, the GlyR α2^V–22L^ missense variant was predicted to be cleaved between amino acids 21 and 22: TNH-FR (Figure 1A), which could influence the efficiency of signal peptide cleavage and cell-surface expression. By contrast, GlyR α2^N38K^ and GlyR α2^K213E^ lie within the ligand-binding ECD (Figure 1A), while GlyR α2^T269M^ affects a highly conserved residue within the pore-forming M2 domain (Figure 1B). Interestingly, substitutions at the equivalent residues to GlyR α2^N38K^, α2^K213E^ and α2^T269M^ have not been observed in the GlyR α1 subunit in startle disease (Chung et al., 2010; Bode et al., 2013; Zhang et al., 2016; Figure 1).

**FIGURE 2:**
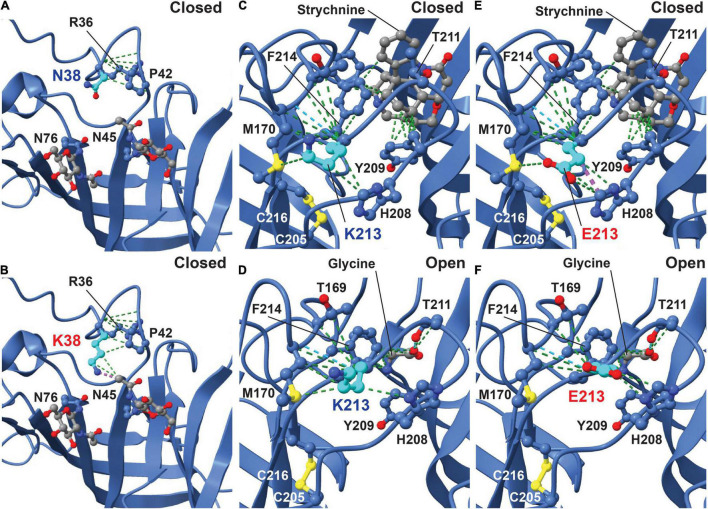
Molecular modeling of the impacts of GlyR α2^N38K^ and α2^K213E^ variants. The potential effects of GlyR α2^N38K^ and GlyR α2^K213E^ variants were visualized using the GlyR α2β pentamer in the strychnine-bound closed state (RCSB: 7L31) and glycine-bound open state (RCSB: 5BKF) (Yu et al., 2021). In the closed state **(A)**, GlyR α2 N38 makes contacts (green lines) with neighboring residues R36 and P42, while mutant GlyR α2^N38K^ **(B)** is predicted to maintain the original contacts but results in clashes (purple lines) with the glycan at residue N45. In the closed state **(C)**, GlyR α2^K213^ makes numerous contacts with neighboring residues (green lines), stabilizing the cysteine loop structure formed by C205 and C216. However, in the open state **(D)**, GlyR α2^K213^ forms stabilizing contacts with the loop and the ligand-binding residue F214 (green lines). In the closed state **(E)**, mutant GlyR α2^K213E^ is predicted to clash with H208 on the opposing side of the cysteine loop (purple lines). Interestingly, in the open state **(F)** the GlyR α2^K213E^ variant increases the number of contacts with neighboring residues, but does not show a clash with H208, suggesting that the open state may be favored for α2^K213E^.

Initial analysis of the potential effects of GlyR α2^N38K^ and GlyR α2^K213E^ variants was conducted using the GlyR α2β pentamer in the closed (RCSB: 7L31) and glycine-bound open state (RCSB: 5BKF) (Yu et al., 2021). GlyR structures were visualized using the UCSF ChimeraX molecular visualization program (Pettersen et al., 2021). The GlyR α2^N38K^ variant introduces a larger charged side-chain that is predicted to result in clashes with the glycan attached to residue N45 in both closed and open states (Figures 2A,B; Yu et al., 2021). Artificial mutations of the corresponding consensus glycosylation site in GlyR α1 (N-X-T), encompassing N38 and S40, have been found to be essential for GlyR homo-oligomerization and receptor biogenesis (Griffon et al., 1999). However, GlyR α2 is now known to be glycosylated at two sites, N45 and N76 (Yu et al., 2021; Figure 1), with the second site being specific to GlyR α2. We therefore predict that while GlyR α2^N38K^ may not interfere with N-linked glycosylation at N76, it could negatively impact GlyR homo-oligomerization and cell-surface trafficking by interfering with glycosylation at N45. GlyR α2^K213^ is located in the second dicysteine loop in the ECD and is flanked by key ligand-binding residues including GlyR α2 Y209, T211 and F214 (Figure 1, blue lettering; Figures 2C,D). GlyR α2^K213E^ introduces charge swap to the region and some loss of flexibility in the side chain. In the closed state, we found an obvious clash with H208 (Figure 2E), but in the open state, the glutamic acid side chain was free of clashes and made additional contacts with Y209 (Figure 2F). These changes suggest that for GlyR α2^K213E^, the open state may be favored, resulting in prolonged opening of the ion channel.

### Molecular Dynamics Simulations of the GlyR α2^T269M^ Variant

**FIGURE 3:**
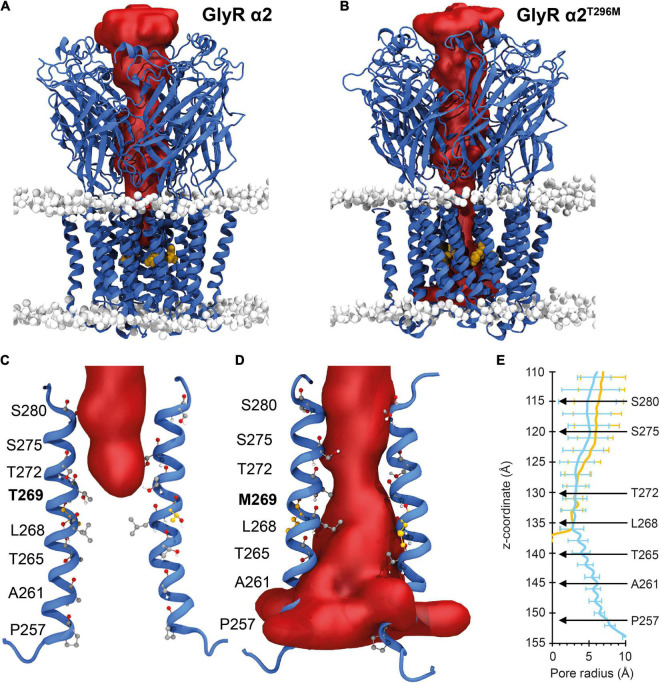
Molecular dynamic simulations of wild-type GlyR α2 and GlyR α2^T269M^ variants. Mean internal cavity surface detected for GlyR α2 homomers in a model membrane (headgroups shown in gray) for: **(A)** wild-type GlyR α2^T269^ and **(B)** the GlyR α2^T269M^ variant over the 1.5 μs of combined production simulation. The site of the M269 mutation is shown in gold. The channel radius (Å) of the wild-type GlyR α2 is compared to the GlyR α2^T269M^ variant over the 1.5 μs of combined production simulation. **(C,D)** The red surfaces show the solvent volume within wild-type GlyR α2^T269^ and GlyR α2^T269M^ channels. Wild-type GlyR is occluded to water at the level of T272, while water permeates the length of the GlyR α2^T269M^ variant. **(E)** The radius of the channel (Å) along the transmembrane region of the longitudinal channel axis is given in the right panel. Selected residues along the channel have been noted for reference and to enable calibration of the distance along the *z*-axis (in Angstroms) with a residue number. blue indicates GlyRα2*^T269^*, yellow indicates GlyRα2*^T269M^*.

**FIGURE 4:**
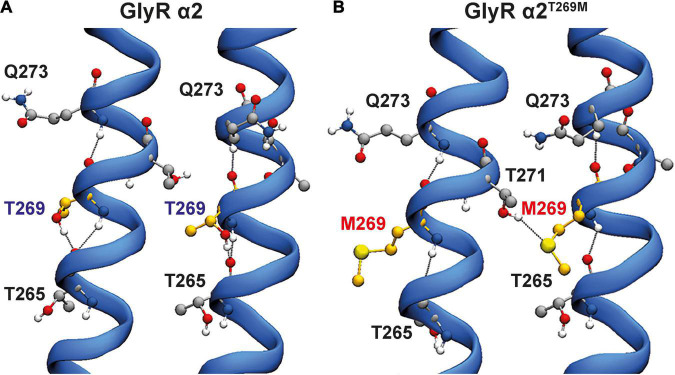
Hydrogen bonding interactions for wild-type GlyR α2 and GlyR α2^T269M^ variants. Two adjacent M2 helices are shown and surrounding amino acids showing interactions between adjacent M2 domains. **(A)** In the wild-type GlyR α2, T269 is stabilized by hydrogen bonds with the adjacent polar residues T265 and Q273 located in the same M2 domain in an arrangement where the side chains are stacked. **(B)** Conversely, while the larger, non-polar mutant M269 also interacts with T265 and Q273, the longer side-chain forms an additional interaction with T271 in the M2 domain of the adjacent GlyR α2 monomer.

For the GlyR α2^T269M^ variant, we used molecular dynamics simulations to examine the potential effects of this substitution in the M2 domain. Wild-type GlyR α2^T269^ and mutant α2^M269^ GlyR homopentamers were stable over the triplicate 500 ns simulations (Figures 3A–D). The ion channel for the GlyR α2 homopentamer is closed around T272, giving a physical occlusion to the channel pore (Figures 3A,C,E). By contrast, the M269 ion channel is open and allows movement between the extracellular and intracellular solutions (Figures 3B,D,E). This opening of the channel leads to an increase in the channel volume for the GlyR α2^M269^ vs. wild-type GlyR α2^T269^ and a corresponding increase in the number of water molecules within the channel. An average of 56 water molecules was found within the channel for wild-type GlyR α2^T269^ across each 500 ns replicate simulation, compared to an average of 130 water molecules for the GlyR α2^M269^ variant. The changes in ion channel volume and water occupancy were also coupled with an increase in Cl^–^ ion presence in the channel for GlyR α2^M269^, compared to wild-type GlyR α2^T269^. Here, one or more Cl^–^ ions are found in the channel for 27% of the total 1,500 ns of combined simulation time for α2^M269^, compared to only 5% of the total combined simulation time of the wild-type α2^T269^ system. The changes in ion channel properties between the wild-type α2^T269^ and α2^M269^ systems are due to altered inter-residue interactions in this region of the channel (Figures 4A,B). Specifically, in the wild-type GlyR α2, T269 is stabilized by hydrogen bonds with the adjacent polar residues T265 and Q273 located on the same M2 domain in an arrangement where the side chains are stacked (Figure 4A). Conversely, while the larger, non-polar mutant M269 also interacts with T265 and Q273, the longer side chain forms an additional interaction with T271 in the M2 domain of the adjacent GlyR α2 monomer (Figure 4B). When T271 interacts with the mutated M269, T271 is no longer able to stabilize the closed conformation of the L268 gating residue. In the GlyR α2^M269^ system, the backbone of L268 forms hydrogen bonds with the sidechain of T272. Collectively, these changes in hydrogen bonding in the region surrounding M269 and L268 predict an opening of the channel for GlyR α2^M269^ compared to wild-type α2^T269^, increasing both the water and Cl^–^ occupancy of the channel.

### GlyR α2^V–22L^, α2^N38K^, α2^K213E^ and α2^T269M^ Variants Exhibit Impaired Cell-Surface Trafficking

**FIGURE 5:**
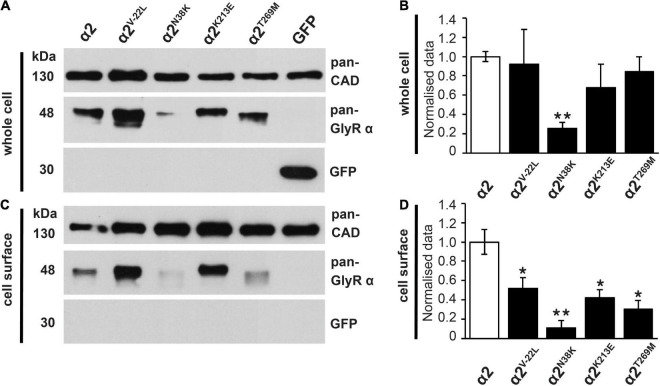
Whole-cell protein and cell-surface expression of GlyR α2 subunit variants. **(A,C)** Whole-cell protein and cell-surface protein fractions from HEK293 cells transfected with either wild-type GlyR α2 or GlyR α2 variants α2^V–22L^, α2^N38K^, α2^K213E^, or α2^T269M^ were immunostained for GlyR α2 using the pan-GlyR α subunit antibody mAb4a (48 kDa). For cell-surface biotinylation assays, cells were also transfected with EGFP as an internal control to ensure that only cell-surface proteins (30 kDa) were isolated. Cadherin served as housekeeping protein for both whole-cell and cell-surface expression and was detected by a pan-cadherin (pan-CAD) antibody (130 kDa). **(B,D)** Quantification of whole-cell and cell-surface protein fractions, normalized to pan-cadherin. The expression of wild-type GlyR α2 subunit was set to 1 (reflecting 100%). We noted a significant reduction of cell-surface protein for all GlyR α2 variants compared to the wild-type GlyR α2 control; significance values are *p < 0.05, **p < 0.01. All results are detailed in Table 1.

To examine the effects of these GlyR α2 variants on cell-surface expression, we measured whole-cell versus surface GlyR expression levels by labeling of surface proteins with biotin followed by cell lysis and precipitation of biotin-labeled proteins using streptavidin beads (Figures 5A–D and Table 1). Samples from the lysate refer to the whole-cell protein pool (Figures 5A,B), samples of biotinylated proteins refer to the surface-expressed receptor protein (Figures 5C,D). Whole-cell and surface-expressed protein levels were first normalized to the expression levels of cadherin, and then relative to wild-type GlyR α2 levels which were designated as 100%. While whole-cell expression of the signal peptide variant GlyR α2^V–22L^ was not significantly reduced compared to wild-type GlyR α2, cell-surface expression was significantly reduced (α2^V–22L^ 52 ± 12% of control values, *p < 0.05). By contrast, for GlyR α2^N38K^, predicted to interfere with N-linked glycosylation, both whole-cell and cell-surface expression levels were significantly reduced (α2^N38K^ whole-cell 26 ± 6%^**^; cell surface 11 ± 7%^**^; ^**^p < 0.01, Figures 5A–D and Table 1). For the remaining two GlyR α2 variants α2^K213E^ and α2^T269M^ whole-cell expression levels were indistinguishable from wild-type GlyR α2 (α2^K213E^ 68 ± 25% and α2^T269M^ 86 ± 15% of wild-type values), while both showed diminished cell-surface expression (α2^K213E^: 42 ± 8%*; α2^T269M^: 30 ± 9%*; *p < 0.05, Figures 5A–D and Table 1). Hence, all GlyR α2 missense variants affected cell-surface expression to varying degrees, but none were completely retained in the endoplasmic reticulum or other subcellular compartments.

**TABLE 1:** Cellular expression profiles of GlyR α2 ASD/DD variants expressed in HEK293 cells.

	Whole cell		Cell-surface	
Construct	Relative expression	Normalized expression (%)	Relative expression	Normalized expression (%)
GlyR α2 wild-type	0.54 ± 0.03	100 ± 5	0.63 ± 0.08	100 ± 12
GlyR α2^V–22L^	0.49 ± 0.19	92 ± 36	0.32 ± 0.07	52 ± 12*
GlyR α2^N38K^	0.14 ± 0.03	26 ± 6**	0.07 ± 0.04	11 ± 7**
GlyR α2^K213E^	0.37 ± 0.13	68 ± 25	0.26 ± 0.05	42 ± 8*
GlyR α2^T269M^	0.46 ± 0.08	86 ± 15	0.19 ± 0.06	30 ± 9*

Relative expression reflects the expression values obtained for the GlyR variants in relation to levels of the control protein pan-cadherin. For normalized expression, expression of GlyR α2 variants is shown as a percentage of wild-type GlyR α2 subunit values (100%). p-values were calculated relative to wild-type GlyR α2 homomers using Student’s t-test (analysis of variance) and values below *p < 0.05 were considered significant, **p < 0.01. Values are displayed as means ± standard error of the mean (±SEM).

### Electrophysiological Properties of GlyR α2^N38K^, α2^K213E^ and α2^T269M^ Homomers

**FIGURE 6:**
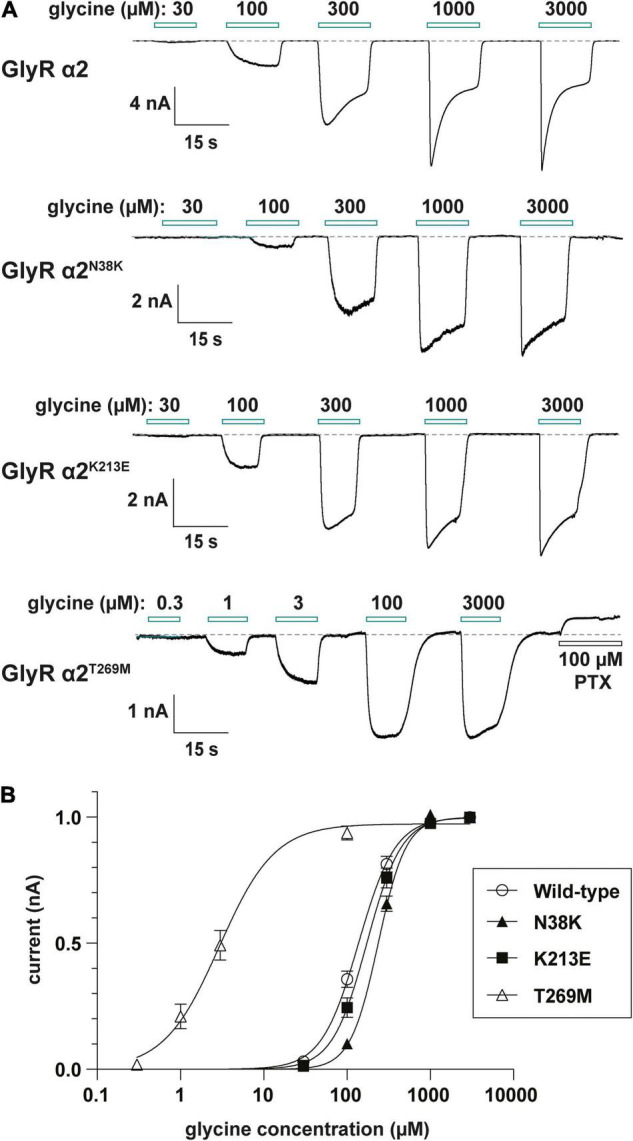
Functional analysis of human GlyR α2 variants using whole-cell patch-clamp electrophysiology. **(A)** Glycine dose-response sample traces for wild-type GlyR α2 and α2^N38K^, α2^K213E^ and α2^T269M^ variants. Note that the GlyR α2^V–22L^ variant was not studied, since this change is in the signal peptide, and is not located in the mature GlyR α2 subunit polypeptide. Horizontal bars indicate the applied glycine concentration in micromolar. The effect of applying 100 μM picrotoxin on baseline current is also shown for the GlyR α2^T269M^ variant. Note that this results in an apparent outward current, reflecting a significant leakage current caused by spontaneous GlyR activity. **(B)** Normalized, averaged glycine dose-response results for wild-type GlyR α2 and α2^N38K^, α2^K213E^ and α2^T269M^ variants. Note that GlyR α2^T269M^ also exhibits a significantly reduced glycine EC_50_ value, which results in GlyR α2^T269M^ activation at low micromolar glycine concentrations. Parameters of best fit to the Hill equation are summarized in Table 2.

Consistent with cell-surface trafficking data, GlyR α2^N38K^ subunit homomers expressed in HEK293 cells exhibited a significantly reduced mean I_max_ value (α2^N38K^ 3.3 ± 0.7 vs. wild-type 8.9 ± 1.6 nA; *n* = 7 cells each; *p* < 0.01) and a significantly increased glycine EC_50_ value (α2^N38K^ 243 ± 12 vs. wild-type 141 ± 14 μM; *n* = 7 cells each; *p* < 0.001) compared to wild-type GlyR α2 subunit homomers (Figures 6A,B and Table 2). Again, this is consistent with a *loss-of-function* for GlyR α2^N38K^. By contrast, despite the reduced expression levels observed in cell-surface biotinylation experiments, GlyR α2^K213E^ subunit homomers exhibited no significant change in either I_max_ or EC_50_ relative to the wild-type GlyR α2 subunit homomers (Figures 6A,B and Table 2), suggesting that this missense change has a more subtle effect on GlyR function, as predicted by molecular modeling. However, as suggested by the location of p.T269M substitution in the ion-channel pore, and molecular dynamics simulations, GlyR α2^T269M^ subunit homomers exhibited a dramatic phenotype (Figures 6A,B and Table 2). GlyR α2^T269M^ homomers not only displayed robust glycine-gated currents but also exhibited a significant leakage current, as revealed by the block of the baseline current by 100 μM picrotoxin, an inhibitor of homomeric GlyRs. Averaged from five cells, the mean magnitude of the picrotoxin-blocked current was 240 ± 35 pA, and the relative magnitude of leak current to saturating whole-cell current in individual cells was 18.7 ± 3.8% (*n* = 5 cells). By contrast, we did not observe any upward deflection in the baseline current when 100 μM picrotoxin was applied to cells expressing wild-type GlyR α2, α2^N38K^ or α2^K213E^ (data not shown).

**TABLE 2:** Properties of wild-type and mutant GlyRs measured using whole-cell patch-clamp electrophysiology in HEK293 cells.

Construct	I_max_ (nA)	n*_*H*_*	EC_50_ (μM)	*n*
GlyR α2 wild-type	8.9 ± 1.6	2.1 ± 0.1	141 ± 14	7
GlyR α2^N38K^	3.3 ± 0.7**	2.8 ± 0.1	243 ± 12***	7
GlyR α2^K213E^	5.2 ± 0.7	2.3 ± 0.3	177 ± 9	7
GlyR α2^T269M^	2.1 ± 0.4***	1.4 ± 0.2	4.5 ± 1.7***	7

The averaged maximal currents (I_max_), Hill coefficients (n_H_), and EC_50_ values in response to glycine activation are shown. p-values were calculated relative to wild-type GlyR α2 homomers using one-way ANOVA followed by Tukey’s post hoc test: **p < 0.01, ***p < 0.001.

Relative to wild-type GlyR α2 subunit homomers, GlyRs containing the α2^T269M^ subunit also exhibited a significantly reduced mean glycine-activated I_max_ current (2.1 ± 0.4 vs. 8.9 ± 1.6 nA; *n* = 7 cells; *p* < 0.001, Table 2) but this was counterbalanced by a significantly reduced glycine EC_50_ value (α2^T269M^ 4.5 ± 1.7 vs. wild-type 141 ± 14 μM; *n* = 7 cells; *p* < 0.001, Figures 6A,B and Table 2). Thus, despite a reduction in cell surface-trafficking, the leak current and the high glycine sensitivity of GlyR α2^T269M^ subunit homomers are suggestive of a *gain-of-function* that is predicted to increase glycinergic signaling at synapses. It should be noted that tonic leak currents that impair cell viability have been previously observed for “leaky” GlyR α1 subunit mutants (Bode et al., 2013; Zhang et al., 2016). The degree of degradation in viability may well have been proportional to the functional expression level of the GlyR α2^T269M^ construct in individual cells. Thus, by selecting relatively healthy cells for analysis, we may have biased cell selection toward weakly expressing cells with smaller than average whole-cell current magnitudes.

### Properties of GlyR α2^N38K^, α2^K213E^ and α2^T269M^ Variants in Artificial Synapses

**FIGURE 7:**
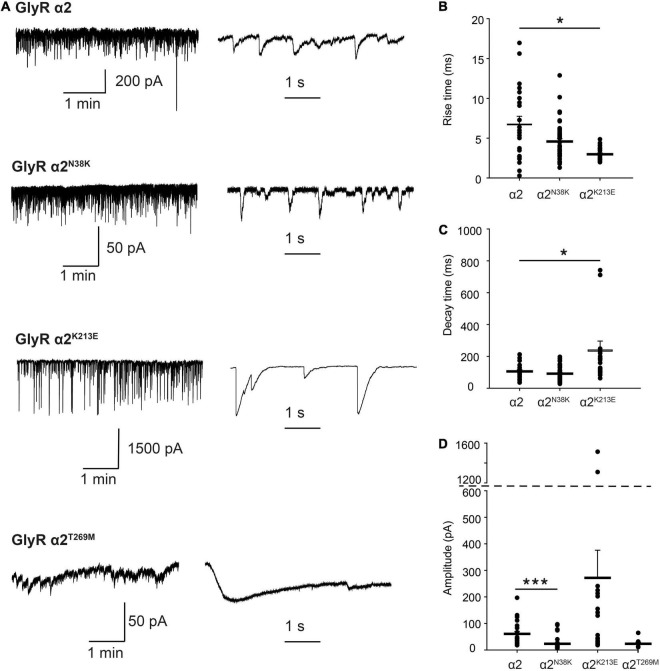
Properties of spontaneous inhibitory postsynaptic currents (IPSCs) recorded from artificial synapses incorporating human GlyR α2 ASD variants. **(A)** Representative recordings of IPSCs from HEK293 cells expressing wild-type GlyR α2 and α2^N38K^, α2^K213E^, and α2^T269M^ variants at two temporal scales. **(B–D)** Mean 10–90% rise times, IPSC decay time constants and amplitudes. Each data point represents the global average of all well-isolated events recorded from a single cell. Means were tested for significance relative to WT using one-way ANOVA followed by Tukey’s *post hoc* test: *p < 0.05, ***p < 0.001. All results are tabulated in Table 3.

For functional studies in artificial synapses, we utilized homomeric α2 subunit GlyRs, as these extrasynaptic GlyRs represent the predominant prenatal isoform that is critical for interneuron migration in the developing cortex (Avila et al., 2013, 2014). In the artificial synapse system, homomeric α2 GlyRs exhibit slow decay time constants, and are thought to be perisynaptic in location due to slow 10–90% rise times (Table 3), implying that they are located a substantial distance from presynaptic terminals (Zhang et al., 2015a). Whole-cell recordings from transfected HEK293 cells in co-culture with spinal neurons exhibited robust, spontaneous IPSCs with amplitudes up to 1000 pA. Sample recordings at low and high temporal resolution for wild-type GlyR α2 and each variant are shown in Figures 7A–D, left and center panels. After each recording, we normalized and digitally averaged all well-separated IPSCs to produce a single globally averaged waveform. We thereby obtained a single averaged 10–90% rise time, decay time constant and amplitude for each cell. Table 3 summarizes the mean values obtained for each of the three parameters. These values were averaged from 9 to 46 cells as indicated. IPSCs mediated by wild-type GlyR α2 exhibited a mean amplitude of 60.6 ± 9.7 pA, a 10–90% rise time of 6.76 ± 0.98 ms and a mean decay time constant of 105.3 ± 11.4 ms (*n* = 22 cells). These values are very similar to those recorded previously from wild-type GlyR α2 expressed in artificial synapses (Zhang et al., 2015b). Relative to wild-type GlyR α2 values, IPSCs mediated by GlyR α2^N38K^ exhibited significantly reduced amplitudes (23.4 ± 3.0 pA; *p* < 0.001, *n* = 46 cells) although IPSC rise and decay times were unchanged (Table 3). Notably, GlyR α2^K213E^-mediated IPSCs were dramatically different from wild-type GlyR α2 values, with significantly larger amplitudes (α2^K213E^ 271.9 ± 104.2 vs. wild-type 60.6 ± 9.7 pA; *n* = 22 and 46 cells, respectively; *p* < 0.001), significantly faster rise times (α2^K213E^ 4.58 ± 0.35 vs. wild-type 6.76 ± 0.98 ms; *n* = 22 and 46 cells, respectively; *p* < 0.05), and significantly slower decay times (α2^K213E^ 240.4 ± 55.4 vs. wild-type 105.3 ± 11.4 ms; *n* = 22 and 46 cells, respectively; *p* < 0.05). Thus, although this variant appeared to have little functional effect in patch-clamp experiments, in artificial synapses GlyR α2^K213E^ dramatically enhanced glycinergic signaling suggesting that it causes a *gain-of-function.* Unfortunately, HEK293 cells expressing GlyR α2^T269M^ were unhealthy when maintained in co-culture for several days and this permitted only short-lasting, unstable recordings. We were able to obtain an estimate of the mean IPSC amplitude (23.2 ± 5.8 pA) from *n* = 9 cells despite attempted recordings from >200 cells. Moreover, due to the extraordinarily long IPSC decay times (e.g., Figure 7D, bottom center panel), it was not possible to isolate individual events, and thus we could not quantify mean IPSC rise and decay times. However, these results are consistent with the leak currents and gain-of-function observed in simple patch-clamp experiments.

**TABLE 3:** Properties of IPSCs mediated wild-type and mutant GlyRs in artificial synapses.

Construct	Amplitude (pA)	Rise time (ms)	Decay time (ms)	*n*
GlyR α2 wild-type	60.6 ± 9.7	6.76 ± 0.98	105.3 ± 11.4	22
GlyR α2^N38K^	23.4 ± 3.0***	4.58 ± 0.35	91.3 ± 6.7	46
GlyR α2^K213E^	271.9 ± 104.2***	2.97 ± 0.18*	240.4 ± 55.4*	23
GlyR α2^T269M^	23.2 ± 5.8	N.D.	N.D.	9

The averaged IPSC peak amplitudes, rise times and decay time constants are shown. p-values were calculated relative to wild-type GlyR α2 homomers using one-way ANOVA followed by Tukey’s post hoc test: *p < 0.05, ***p < 0.001. N.D., not determined.

## Discussion

In this article, we identified functional alterations for four missense variants in *GLRA2*, encoding the GlyR α2 subunit that had previously been associated with human ASD and developmental disorders, using a combination of bioinformatics, molecular dynamics simulations, cellular models of GlyR trafficking and electrophysiology using artificial synapses. The GlyR α2^V–22L^ variant resulted in altered predicted signal peptide cleavage and a reduction in cell-surface expression, suggestive of a partial loss-of-function. GlyR α2^V–22L^ was reported in a female proband with ASD and a verbal IQ of 63 (Iossifov et al., 2014; Supplementary Table 1). Given the alteration in predicted signal cleavage, coupled with a significant reduction in cell-surface expression (52 ± 12% of control values, Figure 5 and Table 1) we suggest that this variant should be classified as *potentially pathogenic*. By contrast, molecular modeling of the GlyR α2^N38K^ variant (Figures 2A,B) revealed that the GlyR α2^N38K^ variant introduces a larger, charged side-chain that is predicted to form contacts with GlyR α2^N45^, which is predicted to be glycosylated *in vivo*. Glycosylation has long been known to be an essential determinant of GlyR maturation and homo-oligomerization (Griffon et al., 1999) and hence GlyR α2^N38K^ is predicted to interfere with N-linked glycosylation, GlyR homo-oligomerization and/or cell-surface trafficking. The latter was demonstrated biochemically by measuring whole-cell and cell-surface expression of GlyR α2^N38K^, revealing a dramatic reduction in both parameters (Figure 5 and Table 1). GlyR α2^N38K^ also showed a reduced mean I_max_ value (α2^N38K^ 3.3 ± 0.7 nA vs. wild-type 8.9 ± 1.6 nA) and a significantly increased glycine EC_50_ value (α2^N38K^ 243 ± 12 μM vs. wild-type 141 ± 14 μM) versus wild-type GlyR α2, again consistent with a *loss-of-function* (Figures 6A,B and Table 2). In artificial synapses, this was reflected in significantly reduced amplitudes of IPSCs mediated by GlyR α2^N38K^ (23.4 ± 3.0 pA vs. 60.6 ± 9.7 pA for wild-type GlyR α2, Figure 7 and Table 3). Curiously, GlyR α2^N38K^ was reported as a *de novo* variant in a male and assigned as a “designated unaffected sibling” to an affected case (Krumm et al., 2015; Supplementary Table 1). However, given our bioinformatic and functional findings, suggesting that this variant is highly deleterious to GlyR α2 function, we would definitely classify the GlyR α2^N38K^ variant as *pathogenic* and would advise the referring clinicians to revisit this case/family.

GlyR α2^K213E^ was reported in a male individual with a refractory epilepsy, microcephaly, and severe developmental delay (Supplementary Table 1). GlyR α2^K213E^ homomers showed a reduction in cell-surface expression (α2^K213E^: 42 ± 8% of wild-type values, Figure 5 and Table 1). However, in whole-cell patch clamp electrophysiology GlyR α2^K213E^ subunit homomers exhibited no significant change in either I_max_ or EC_50_ relative to the wild-type GlyR α2 subunit homomers (Figures 6A,B and Table 2). While this evidence would normally result in this variant being classified as non-pathogenic, high CADD scores, plus molecular modeling findings caused us to reconsider. In particular, GlyR α2^K213E^ introduces change from a positive to a negatively charged side chain in the second dicysteine loop, which contains several ligand-binding residues (GlyR α2 Y209, T211, and F214, Figure 1). In the closed state, we found an obvious clash with H208, but in the open state, we found that the α2^K213E^ side chain was free of clashes and made additional contacts with Y209. These changes suggested that the open state may be favored for this mutant, resulting in prolonged channel opening. This theory was borne out in artificial synapse experiments, where we observed that IPSCs mediated by α2^K213E^ had significantly larger amplitudes, faster rise times and significantly slower decay times than wild-type GlyR α2 (Figure 7 and Table 3). We therefore classify α2^K213E^ as a *pathogenic gain-of-function* variant that is likely to enhance glycinergic signaling in the developing brain.

Lastly, GlyR α2^T269M^ was reported in a female proband in the Deciphering Developmental Disorders Study (2017). It has previously been suggested that GlyR α2 missense mutations in females cannot be associated with ASD since: (i) an intact copy of *GLRA2* is found on the other X chromosome and (ii) because *GLRA2* escapes X-inactivation in the vast majority of tissues including the brain (Cotton et al., 2015). However, this assumption is clearly incorrect as exemplified by our previous study of the GlyR α2^R323L^ mutation found in a female proband (Zhang et al., 2017). GlyRs form either homomeric (5α) or heteromeric complexes (4α:1β) *in vivo*, so mutant GlyRs subunits can incorporate into GlyRs alongside wild-type subunits. GlyR α2^T269M^ homomers showed diminished cell-surface expression (Figure 5 and Table 1). Consistent with this finding, in whole-cell recordings where glycine was applied under steady-state conditions, GlyRs containing the α2^T269M^ subunit had a significantly decreased mean glycine-activated I_max_ current (α2^T269M^ 2.1 ± 0.4 vs. wild-type 8.9 ± 1.6 nA). However, this was counterbalanced by a significantly increased sensitivity to glycine (EC_50_ values α2^T269M^ 4.5 ± 1.7 vs. wild-type 141 ± 14 μM; *n* = 7 cells; *p* < 0.001). As predicted from our molecular dynamics simulations (Figures 3, 4), GlyR α2^T269M^ homomers also exhibited a significant leakage current that could be revealed by blockade with 100 μM picrotoxin (Figure 6A). Averaged from five cells, the mean magnitude of the picrotoxin-blocked current was 240 ± 35 pA. This mutant was particularly difficult to study in artificial synapses, as HEK293 cells expressing GlyR α2^T269M^ were unhealthy when maintained in co-culture. Despite this, an estimate of mean IPSC amplitude (23.2 ± 5.8 pA) was obtained. It is also noteworthy that spontaneous IPSC decay rates were dramatically prolonged (Figure 7) as previously observed with other GlyR mutants that reduce the glycine EC_50_ (Dixon et al., 2015; Zhang et al., 2016). Based on these results, we classify α2^T269M^ as a *pathogenic alteration-of-function* variant (given the reduced glycine EC_50_ plus leak current) that is predicted to enhance glycinergic signaling in the developing brain. It is also noteworthy that the GlyR α2^T269M^ mutation has recently been reported as a *de novo* mutation in six additional female subjects (Marcogliese et al., 2022), making it the first recurrent *GLRA2* pathogenic mutation. Using a novel *Drosophila*-based functional system for ASD mutations, Marcogliese et al. (2022) also classified GlyR α2^T269M^ as a *gain-of-function* allele based on experiments overexpressing human GlyR α2^T269M^ in pre-synaptic photoreceptors and postsynaptic neurons, reporting a significant increase in amplitudes of “OFF” transients for the GlyR α2^T269M^ transgenic line. This artificial system has severe limitations for the study of GlyR α2 subunit mutants, since glycinergic neurons in *Drosophila* seem to be limited to small ventral lateral neurons (sLNvs) involved in circadian behavior (Frenkel et al., 2017). It is therefore unclear how glycine would be released onto exogenous GlyRs expressed in photoreceptors. However, our study has revealed a convincing explanation for the increase in “OFF” transient amplitudes observed by Marcogliese et al. (2022). GlyR α2^T269M^ forms spontaneously opening channels that do not require activation by endogenous glycine.

In summary, our study has revealed that GlyR α2 subunit mutations are a complex mix, or loss, gain and alteration of function, associated with a range of clinical phenotypes. For this reason, we predict that many more *GLRA2* mutations remain to be discovered in a spectrum of neurological disorders encompassing ASD, DD, epilepsy and neuronal migration disorders and that detailed functional characterization will be required to distinguish different mutational pathomechanisms. The comprehensive functional characterization of the GlyR α2^K213E^ and α2^R323L^ variants has also provided a solid basis for the production of knock-in mice that have GlyR α2 *gain-of-function* mutations to examine the effects of enhanced GlyR α2 function on cortical progenitor homeostasis, interneuron migration and other biological roles of this important GlyR subtype.

## Data Availability Statement

The original contributions presented in the study are included in the article/Supplementary Material, further inquiries can be directed to the corresponding author.

## Ethics Statement

Ethical review/approval was not required for this study of de-identified genetic variants in accordance with local legislation and institutional requirements. Written informed consent to participate in the study was provided by the legal guardians for the individual with the GlyR a2p.E213K variant.

## Author Contributions

RH conceived the study. ES, GR, and KS identified the p.K213E mutation in diagnostic exome sequencing. RH, MW, and LD performed the bioinformatic analysis, molecular modeling, and generated GlyR α2 subunit expression constructs and mutants. XC and JL conducted artificial synapse experiments and electrophysiology. NS and CV conducted cell-surface trafficking experiments. KW and MO’M conducted molecular dynamics simulations. RH, CV, JL, and MO’M drafted the manuscript. All authors were involved in revising the manuscript for important intellectual content and gave approval for the final version to be published.

## Conflict of Interest

The authors declare that the research was conducted in the absence of any commercial or financial relationships that could be construed as a potential conflict of interest.

## Publisher’s Note

All claims expressed in this article are solely those of the authors and do not necessarily represent those of their affiliated organizations, or those of the publisher, the editors and the reviewers. Any product that may be evaluated in this article, or claim that may be made by its manufacturer, is not guaranteed or endorsed by the publisher.
